# Metabolomic profile of secondary hyperparathyroidism in patients with chronic kidney disease stages 3–5 not receiving dialysis

**DOI:** 10.3389/fendo.2024.1406690

**Published:** 2024-07-04

**Authors:** Lingling Gan, Lijun Wang, Wanyi Li, Yamei Zhang, Bei Xu

**Affiliations:** ^1^ Department of Clinical Laboratory, Mianyang Central Hospital, School of Medicine, University of Electronic Science and Technology of China, Mianyang, China; ^2^ Department of Nephrology, Mianyang Central Hospital, School of Medicine, University of Electronic Science and Technology of China, Mianyang, China; ^3^ National Health Commission (NHC) Key Laboratory of Nuclear Technology Medical Transformation, Mianyang Central Hospital, Mianyang, China

**Keywords:** amino acids, lipids, ultra performance liquid chromatography-tandem mass spectrometry, untargeted metabolomics, secondary hyperparathyroidism, chronic kidney disease

## Abstract

**Introduction:**

Secondary hyperparathyroidism (SHPT) is a common and serious complication of chronic kidney disease (CKD). Elucidating the metabolic characteristics of SHPT may provide a new theoretical basis for its prevention and treatment. This study aimed to perform a metabolomic analysis of SHPT in patients with CKD stages 3–5 not receiving dialysis.

**Methods:**

A total of 76 patients with CKD, 85 patients with CKD-SHPT, and 67 healthy controls were enrolled in this study. CKD was diagnosed according to the criteria specified in the Kidney Disease Improving Global Outcomes 2012 guidelines. SHPT was diagnosed by experienced clinicians according to the Renal Disease Outcomes Quality Initiative Clinical Practice Guidelines. Serum renal function markers and the lipid profile were analyzed. Untargeted ultra performance liquid chromatography-tandem mass spectrometry was used to analyze the serum metabolites of patients with CKD and SHPT. Multivariate analysis of the data was performed using principal component analysis and partial least square discriminant analysis. Serum differential metabolites were identified and further characterized using databases. Pathway enrichment analysis was performed using the Kyoto Encyclopedia of Genes and Genomes database. Correlations between differential metabolites and clinical parameters were determined using the Spearman correlation.

**Results:**

The serum metabolomic profiles of patients with CKD with and without SHPT differed significantly. Differential metabolites were mainly enriched in the top four Kyoto Encyclopedia of Genes and Genomes pathways: phenylalanine, tyrosine, and tryptophan biosynthesis; sphingolipid metabolism; glycerophospholipid metabolism; and phenylalanine metabolism. In total, 31 differential metabolites were identified; of these, L-tryptophan and (R)-(+)-1-phenylethylamine were decreased, while other amino acids and their derivatives, uremia toxins, carnitine, and lipids, were increased significantly in patients with SHPT compared to those without. The 14 lipid metabolites were positively correlated with levels of Urea, serum creatinine, cystatin C, and triglycerides and negatively correlated with the estimated glomerular filtration rate and levels of total and high- and low-density lipoprotein cholesterol.

**Discussion:**

Disturbed amino acid and lipid metabolism were more apparent in patients with SHPT than in those without. This metabolomic profile of SHPT may provide a therapeutic foundation for its future clinical management.

## 1 Introduction

Secondary hyperparathyroidism (SHPT) is a common complication in patients with chronic kidney disease (CKD) and an important component of CKD mineral and bone disorders (1). SHPT can occur in the early stages of CKD, with 40% of patients with CKD stage 3 exhibiting elevated parathyroid hormone (PTH) levels of >65 pg/mL, but becomes more frequent as kidney function decreases, affecting up to 80% of patients in stage 4 (2, 3). SHPT is characterized by hypocalcemia, hyperphosphatemia, parathyroid hyperplasia, and the hypersynthesis and secretion of PTH. Excessive PTH can increase bone resorption and alter the metabolism of calcium and phosphorus, ultimately leading to serious complications, including skeletal lesions, anemia, vascular calcification, and cognitive impairment, which increase the risk of all-cause mortality (4, 5). Recently, SHPT has been independently associated with CKD progression and the incidence of cardiovascular events in patients with CKD (6). Therefore, effective management of SHPT is critical; however, this remains challenging. For patients with CKD receiving dialysis, the target range of PTH is set at 2~9 times the upper normal limit. Calcimimetics, calcitriol, and/or active vitamin D analogs (alone or in combination) have been used to treat SHPT in patients on hemodialysis. When PTH-lowering therapies fail, parathyroidectomy remains one of the most effective means of treating SHPT. By contrast, the optimum management of SHPT in patients with CKD not receiving dialysis is not as clearly understood, and there is currently no established target PTH range. Recently, it has also been shown that physicians treating these patients have poor knowledge of mineral and bone disorder management (7). Therefore, SHPT seriously affects the quality of life of patients with CKD, places a high economic burden on individuals and society, and is challenging to treat and poorly understood (8, 9). Elucidating its metabolic profile may offer new insights into the management of SHPT in patients with CKD not receiving dialysis.

Metabolomics analyzes metabolites to assess their relationship with physiological and pathological changes. Currently, liquid chromatography-mass spectrometry (MS), a sensitive and high-throughput technique, is considered the best method for precise metabolomics (10). Previous studies have investigated the metabolic features of SHPT. Wu et al. identified a varied pattern of endogenous metabolites in patients with SHPT, comparing those with PTH levels >300 and 150–300 pg/mL; 30 metabolites were elevated in CKD mineral and bone disorders (11). Another investigation focused on the metabolic profile of patients with PTH >600 pg/mL before parathyroidectomy and forearm transplantation and those with PTH <150 pg/mL postoperatively and identified five metabolites with a moderate to strong correlation with PTH (12). Despite these studies, current understanding of CKD-SHPT remains insufficient; in particular, the serum metabolic profile of patients with CKD-SHPT not undergoing dialysis has not been investigated. Therefore, this study used an untargeted ultra performance liquid chromatography (UPLC)-tandem MS-based metabolomics approach to analyze sera from patients with CKD and SHPT not receiving dialysis. The differential metabolites and their metabolic pathways were analyzed to identify the endogenous metabolic characteristics of patients with CKD and SHPT.

## 2 Materials and methods

### 2.1 Study design and participants

A total of 161 patients with CKD stages 3−5 but not undergoing dialysis, who were admitted to our hospital between January 2020 and December 2022 were enrolled in the study. Of these, 85 had SHPT (CKD-SHPT group) and 76 did not (CKD group). The patients in CKD and CKD-SHPT groups were matched for kidney function, as determined by estimated glomerular filtration rate (eGFR). The control group consisted of 67 healthy individuals who were undergoing a physical examination (HC group). The participants in all groups were matched for age and sex. The study was approved by the Ethical Review of Medical Technology Committee of Mianyang Central Hospital (approval number: S-2021-003), and complied with all relevant national regulations and institutional policies. The study was conducted in accordance with the principles outlined in the Declaration of Helsinki.

The inclusion criteria were as follows (1): diagnosis of CKD caused by primary chronic glomerulonephritis (2); no evidence of inflammatory, neoplastic, or infectious diseases, uncontrolled hypertension, cardiac insufficiency, neurological or psychiatric dysfunction, or severe bleeding disorders; and (3) aged >18 years.

The exclusion criteria were as follows (1): incomplete clinical data (2); receiving dialysis (3); history of thyroid or parathyroid disease, blood transfusion, nephrectomy, or kidney transplantation (4); presence of diabetes, hypertension, malignant tumors, immune system diseases, cardiovascular or neurological diseases, other endocrine system diseases, or recent infection-related symptoms (5); presence of serious or poorly controlled medical conditions (6); pregnancy or lactation; and (7) aged <18 years.

CKD was diagnosed in accordance with the criteria set out in the “Kidney Disease Improving Global Outcomes 2012 Clinical Practice Guideline for the Evaluation and Management of Chronic Kidney Disease (13).” The eGFR was calculated using a modified modification of diet in renal disease equation (14) and used to determine the severity of CKD based on Kidney Disease Improving Global Outcomes criteria: stage 3, eGFR 30–59 mL/min/1.73 m^2^; stage 4, eGFR 15–29 mL/min/1.73 m^2^; stage 5, eGFR <15 mL/min/1.73 m^2^.

SHPT was diagnosed by experienced clinicians according to the Kidney Disease Outcomes Quality Initiative Clinical Practice Guidelines. The normal reference range of PTH is 10−65 pg/mL, and the levels of PTH in the various stages of CKD with SHPT were higher than the normal range values. The levels of PTH exceeded 70 pg/mL in stage 3 CKD, 110 pg/mL in stage 4 CKD, and 300 pg/mL in stage 5 (15).

### 2.2 Sample collection and measurement of laboratory indicators

Clinical information, including data on sex, age, and routine biochemical analyses, were collected. Peripheral venous blood (5 mL) was drawn from all subjects in the morning after an 8–12-h overnight fast. The serum was assayed for levels of intact PTH (1−84), albumin, urea, serum creatinine, cystatin C, calcium, phosphorus, total cholesterol (TC), triglycerides (TG), high-density lipoprotein cholesterol (HDL-C), and low-density lipoprotein cholesterol (LDL-C), and eGFR was calculated, using a fully automated Cobas E801 analyzer (Roche Diagnostics Corporation, Indianapolis, IN, USA). A separate serum sample was reserved for metabolomics.

### 2.3 Metabolomics

As previously described (16, 17), serum was removed from the -80°C freezer and slowly thawed at room temperature. Clenbuterol and chloramphenicol were used as internal standards; 5 μL of these were mixed with 100 μL serum and 400 μL methanol-acetonitrile (1:1 vol/vol), followed by ultrasonic oscillation, incubation for 1 h at -20°C, and centrifugation. The resultant supernatant was passed through a 0.22-μm filtration membrane (Merck Millipore, Burlington, MA, USA); 5 μL was used for metabolomics.

An Agilent^®^ 1290 Infinity II UPLC system (Agilent Technologies, Inc., Santa Clara, CA, USA) coupled with a Triple TOF 5600+ MS system (AB Sciex LLC, Framingham, MA, USA) was used for metabolomics. Sample separation was achieved using an ACQUITY HSS T3 column (100 × 2.1 mm; inner diameter, 1.8 µm; Waters Corporation, Milford, MA, USA).

The mobile phase was composed of 0.1% formic acid in water (solvent A) and 0.1% formic acid in acetonitrile (solvent B), the flow rate was 0.30 mL/min and the column temperature was 30°C. The UPLC-MS/MS analytical conditions were as previously described (18). For MS, both positive and negative electrospray ionization (ESI) modes were used. To guarantee the quality of the nontargeted bioanalytical data, a pooled quality control (QC) sample, prepared by combining 10 μL supernatant from each sample, was injected after every tenth sample.

### 2.4 Metabolomics analysis

Analysis was performed as previously described (18). Principal component analysis and partial least square discriminant analysis (PLS-DA) were used to investigate data clustering trends and differences among the groups.

Accurate metabolite characterization was performed by matching databases. Metabolites with statistical significance (p<0.05), fold change threshold >1.5 or <2/3, and variable importance in the projection >1 were identified. Pathway analysis was performed using the Kyoto Encyclopedia of Genes and Genomes and MetaboAnalyst databases (19).

### 2.5 Statistical analysis

Statistical analyses were performed using SPSS software version 26.0 (IBM Corporation, Armonk, NY, USA). Categorical data are expressed as numbers and percentages and were compared using the chi-squared test. Normally distributed data are expressed as mean ± standard deviation; non-normally distributed data are expressed as median and interquartile range. Continuous normally distributed data were analyzed using the independent samples t-test (two groups) or one-way analysis of variance (> two groups); non-normally distributed data were analyzed using the Mann–Whitney U test (two groups) or Kruskal–Wallis test (> two groups). The correlation between differential metabolites and clinical parameters was determined using the Spearman correlation. Statistical significance was set at p<0.05.

## 3 Results

### 3.1 Patient characteristics

The laboratory data of the participants are presented in **Table 1**. There were no significant differences in sex or age among the three groups, whereas significant differences were observed for all laboratory indices except TC. The CKD and CKD-SHPT groups had similar albumin levels, which were lower than those of the HC group. Urea, serum creatinine, and cystatin C levels were higher in the CKD group than in the HC group and higher in the CKD-SHPT group than in the CKD group. Both calcium and phosphorus levels were significantly higher in the CKD group than in the HC group; calcium levels were decreased and phosphorus levels were further increased in the CKD-SHPT group compared with the CKD group. The CKD-SHPT group had lower TC and higher TG levels than the HC group. Significantly higher levels of TG were observed in the CKD group than in the HC group, but levels did not differ significantly between the CKD and CKD-SHPT groups. HDL-C levels were lower in the CKD group than in the HC group, and lower again in the CKD-SHPT group. LDL-C levels were significantly lower in the CKD-SHPT group than in the HC and CKD groups; there was no significant difference between the HC and CKD groups.

**Table 1:** Demographic characteristics of study population.

Group	HC (*n* = 67)	CKD (*n* = 76)	CKD-SHPT (*n* = 85)	*χ^2^*/*F*/*H*	p
Sex, *n (%)*					
Male	26 (38.8)	40 (52.6)	49 (57.6)	5.540	0.063
Female	41 (61.2)	36 (47.4)	36 (42.4)		
Age, years	58.16 ± 12.89	60.75 ± 16.28	58.45 ± 14.30	0.414	0.662
PTH (pg/mL)	42.55 ± 12.25	39.76 ± 13.03	223.51 ± 131.46^ab^	135.780	< 0.001
Albumin (g/L)	45.93 ± 2.99	38.61 ± 7.50^a^	39.95 ± 4.88^a^	36.487	< 0.001
Urea (mmol/L)	4.66 (3.80-5.76)	12.13 (7.45-22.55)^a^	17.83 (14.58-22.24)^ab^	115.340	< 0.001
Serum Creatinine (μmol/L)	62.40 (56.30-78.20)	244.15 (120.18-556.85)^a^	598.60 (455.60-750.55)^ab^	140.880	< 0.001
Cystatin C (mg/L)	0.83 (0.76-0.98)	2.960 (1.82-5.20)^a^	5.69(4.36-7.30)^ab^	138.650	< 0.001
eGFR (mL/min/1.73 m^2^)	94.30 (81.80-102.50)	27.80 (16.30-45.83)^a^	25.40 (13.40-41.40)^a^	135.573	< 0.001
Calcium (mmol/L)	2.20 ± 0.06	2.33 ± 0.21^a^	2.19 ± 0.22^b^	10.906	< 0.001
Phosphorus (mmol/L)	1.07 ± 0.14	1.32 ± 0.31^a^	1.78 ± 0.51^ab^	66.827	< 0.001
Total cholesterol (mmol/L)	4.42 ± 0.44	4.26 ± 1.29	3.82 ± 2.18^a^	2.926	0.056
Triglycerides (mmol/L)	1.18 (0.87-1.44)	1.32 (0.95-2.18)^a^	1.54 (1.07-2.21)^a^	18.651	< 0.001
HDL-C (mmol/L)	1.40 ± 0.31	1.21 ± 0.40^a^	1.08 ± 0.35^ab^	15.565	< 0.001
LDL-C (mmol/L)	2.76 ± 0.77	2.40 ± 1.05	1.90 ± 0.84^ab^	16.929	< 0.001

a p<0.05 versus HC group.

b p<0.05 versus CKD group. HC, healthy controls; CKD, chronic kidney disease; CKD-SHPT, chronic kidney disease complicated with secondary hyperparathyroidism; eGFR, estimated glomerular filtration rate; HDL-C, high-density lipoprotein cholesterol; LDL-C, low-density lipoprotein cholesterol; PTH, parathyroid hormone.

### 3.2 Multivariate statistical analysis of metabolites

A total of 228 serum samples and 26 QC samples were analyzed in both positive (ESI+) and negative (ESI−) polarity modes. Total ion chromatograms and base peak intensity diagrams are presented in **Supplementary Figures S1A–D**. After data pretreatment (format conversion peak recognition, filtering alignment, and normalization) and quality analysis, a multivariate statistical analysis was performed.

**Figure 1:**
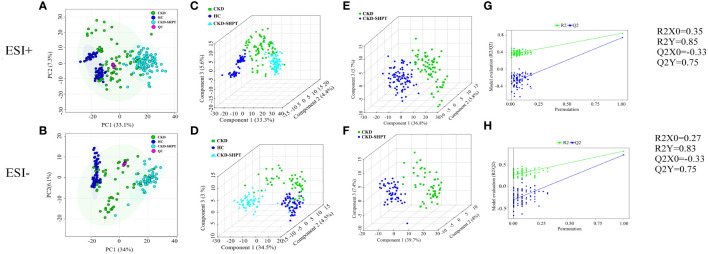
PCA score plots in positive **(A)** and negative **(B)** ion modes among HC, CKD and CKD-SHPT groups. PLS-DA score plots in positive **(C)** and negative **(D)** ion modes among HC, CKD and CKD-SHPT groups. PLS-DA score plots in positive **(E)** and negative **(F)** ion modes between CKD and CKD-SHPT groups. PLS-DA permutation test plots in positive **(G)** and negative **(H)** ion modes between CKD and CKD-SHPT groups. The criterion for evaluating whether there is overfitting in the PLS-DA model is that the regression line at a blue Q2 point crosses or is less than 0 from the abscissa. PCA, principal component analysis; PLS-DA, partial least squares discriminant analysis; HC, healthy controls; CKD, chronic kidney disease; CKD-SHPT, chronic kidney disease complicated with secondary hyperparathyroidism.

The principal component analysis score maps in both ESI− and ESI+ modes reveal tight clustering of the QC samples, indicating high stability and reproducibility of the metabolomics data throughout the analysis (**Figures 1A, B**). However, it failed to distinguish the principal components when comparing the three groups in both ESI− and ESI+ modes.

To select significantly differential metabolites, we performed PLS-DA. As shown in **Figures 1C, D**, significant differences in classification were found among the clustering of the HC, CKD, and CKD-SHPT groups in both ESI+ and ESI− modes. The PLS-DA point cloud map showed clear segregation between the CKD and CKD-SHPT, CKD and HC, and CKD-SHPT and HC groups (**Figures 1E, F**; **Supplementary Figure S2**, **Supplementary Table S1**). To prevent overfitting the PLS-DA models, 100 random permutation experiments were performed. The R2X, R2Y, and Q2 (cumulative) parameters were used to evaluate the PLS-DA model. The Y-intercepts of Q2 distributions had negative values in both ESI+ and ESI− modes in the comparison between the CKD and CKD-SHPT groups, indicating its reliability (**Figures 1G, H**). All these results revealed different metabolic profiles among the three groups.

### 3.3 Screening differential metabolites

**Figure 2:**
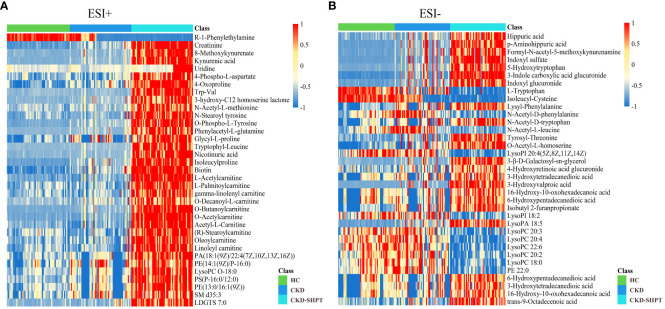
Differential metabolite heat maps in positive **(A)** and negative **(B)** modes. The columns represent samples, the rows represent metabolites, and the relative content of the metabolites is displayed by color. The heat map shows differential metabolites among HC, CKD, and CKD-SHPT groups. HC, healthy controls; CKD, chronic kidney disease; CKD-SHPT, chronic kidney disease complicated with secondary hyperparathyroidism.

A total of 7271 and 5361 peaks were obtained in ESI+ and ESI− modes, respectively. After database matching, 1862 metabolites were identified in ESI+ mode and 1215 in ESI– mode. Upon removal of the exogenous metabolites, the 35 differential metabolites among the HC, CKD, and CKD-SHPT groups were screened out in both ESI+ and ESI− modes according to the following criteria: variable importance in the projection ≥1, fold change ≥1.5 or ≤2/3, and p<0.05. As shown in the heat maps (**Figure 2**), these metabolites exhibited clear clustering.

### 3.4 Enrichment analysis and metabolic pathways of the differential metabolites

**Figure 3:**
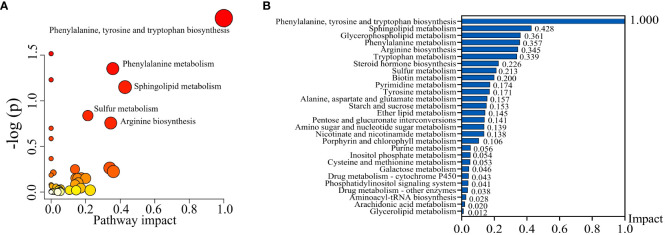
Metabolic pathway analysis. **(A)** Bubble diagram of differential metabolic pathways. **(B)** Histogram of metabolic pathways.

To uncover potential metabolic pathways in SHPT, pathway enrichment and topology analyses were carried out using the MetaboAnalyst database; 28 metabolic pathways were identified. In the bubble diagram (**Figure 3A**), pathway impact values, which indicate the importance of altered metabolites in the respective metabolic pathways, are represented by bubble size; the larger the bubble, the greater the significance. The color indicates –log (p); the darker the color, the smaller the p-value. Disturbed metabolic pathways and their impact values can be seen in the histogram (**Figure 3B**); the top four enriched metabolic pathways were selected. In the comparison of the CKD and CKD-SHPT groups, the most significantly enriched pathway was phenylalanine, tyrosine, and tryptophan biosynthesis, followed by sphingolipid metabolism, glycerophospholipid metabolism, and phenylalanine metabolism (**Table 2**). Significantly enriched metabolic pathways and detailed information on the comparisons between the HC and CKD and HC and CKD-SHPT groups are listed in **Supplementary Table S2**.

**Table 2:** Top four enriched metabolic pathways between groups.

Comparison	Pathway name	KEGG.id	-log(p)	Impact	Hits
CKD vs. CKD-SHPT	Phenylalanine, tyrosine and tryptophan biosynthesis	hsa00400	1.91	1.00	2
	Sphingolipid metabolism	hsa00600	1.15	0.43	5
	Glycerophospholipid metabolism	hsa00564	0.22	0.36	5
	Phenylalanine metabolism	hsa00360	1.35	0.36	3

CKD, chronic kidney disease; CKD-SHPT, chronic kidney disease complicated with secondary hyperparathyroidism; KEGG, Kyoto Encyclopedia of Genes and Genomes.

### 3.5 Differential metabolite analysis

**Figure 4:**
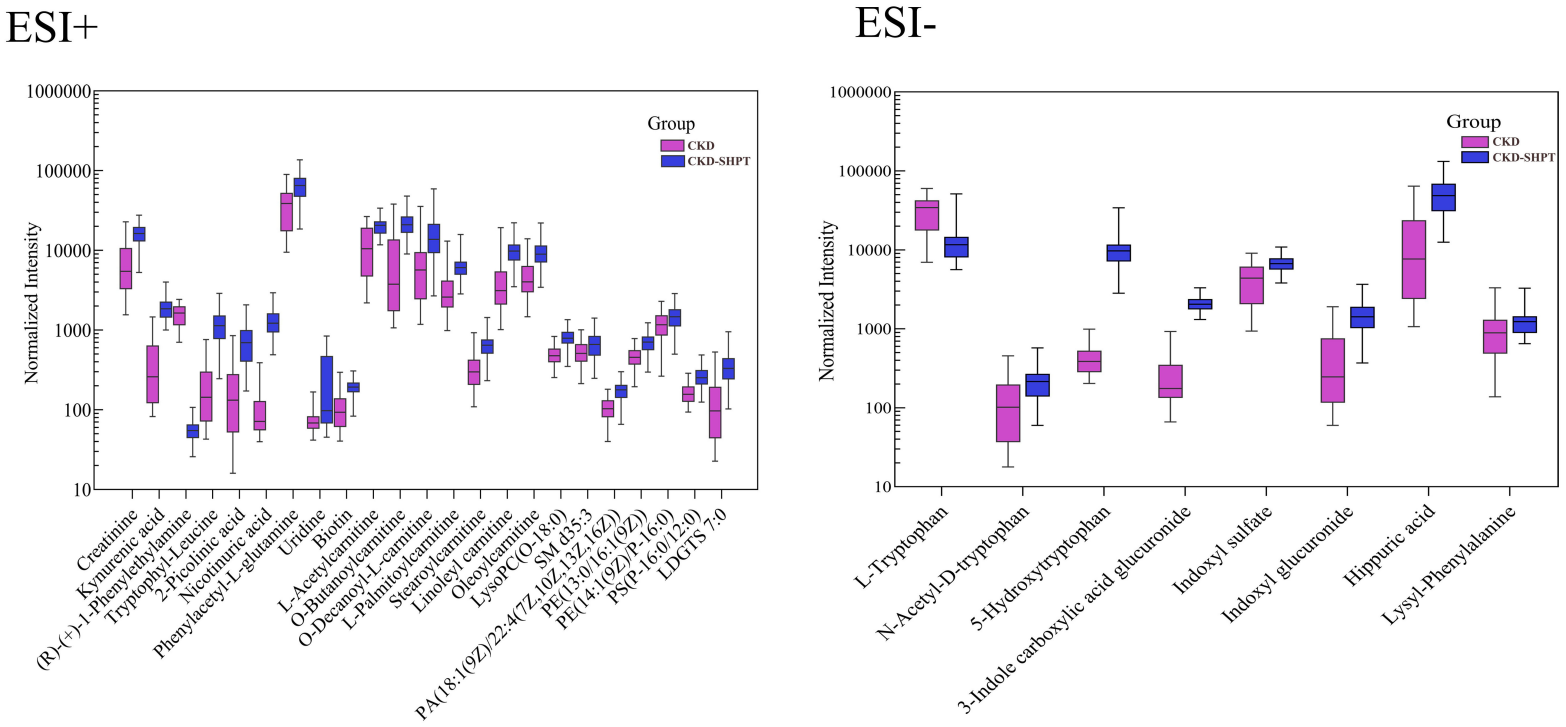
Normalized peak intensity of 31 representative differential metabolites between the HC and CKD-SHPT groups. CKD, chronic kidney disease; CKD-SHPT, chronic kidney disease complicated with secondary hyperparathyroidism.

A total of 31 metabolites met the criteria for significant differences between the CKD and CKD-SHPT groups (**Table 3**). These differential metabolites mainly included uremic toxins, amino acids and their derivatives, carnitine, and lipids. Of the 31 differential metabolites, 29 were increased and two were decreased in the CKD-SHPT group compared with the CKD group. The two downregulated metabolites, L-tryptophan and (R)-(+)-1-phenylethylamine, were amino acids and their derivatives, the levels of other amino acids and their derivatives were increased. Similarly, all uremic toxins, carnitine, and lipids were upregulated in the CKD-SHPT group compared with the CKD group. The normalized intensity peak areas of the selected differentials in ESI+ and ESI− modes are shown in **Figure 4**.

**Table 3:** Significantly differential metabolites between CKD and CKD-SHPT groups.

Metabolites	m/z	Rt(s)	Scan mode	FC	VIP	p	Trend(SHPT)
L-Tryptophan	203.083	4.536	ESI−	0.42	1.12	2.36E-11	↓^***^	
N-Acetyl-D-tryptophan	267.085	6.548	ESI−	1.86	1.14	6.93E-20	↑^***^	
5-Hydroxytryptophan	439.161	5.495	ESI−	3.44	1.06	1.43E-11	↑^***^	
Tryptophyl-Leucine	318.191	4.376	ESI+	4.86	1.14	6.93E-20	↑^***^	
2-Picolinic acid	106.037	5.041	ESI+	4.37	1.12	8.63E-18	↑^***^	
Nicotinuric acid	181.060	4.789	ESI+	4.10	1.22	4.40E-23	↑^***^	
Lysyl-Phenylalanine	292.164	7.747	ESI−	1.72	1.45	4.71E-05	↑^***^	
(R)-(+)-1-Phenylethylamine	122.097	4.767	ESI+	0.09	1.03	1.68E-09	↓^***^	
Phenylacetyl-L-glutamine	265.118	4.963	ESI+	3.48	1.03	3.96E-16	↑^***^	
3-Indole carboxylic acid glucuronide	336.073	5.093	ESI−	3.18	1.32	1.24E-20	↑^***^	
Indoxyl sulfate	425.012	5.065	ESI−	3.93	1.20	1.97E-16	↑^***^	
Indoxyl glucuronide	308.078	4.578	ESI−	2.92	1.03	1.89E-11	↑^***^	
Kynurenic acid	190.050	4.710	ESI+	3.25	1.17	7.33E-19	↑^***^	
Hippuric acid	178.051	5.022	ESI−	3.68	1.12	7.15E-13	↑^***^	
Creatinine	114.067	0.950	ESI+	2.41	1.26	8.03E-26	↑^***^	
Uridine	245.078	7.157	ESI+	3.69	1.10	2.26E-10	↑^***^	
Biotin	245.095	4.256	ESI+	1.99	1.20	1.02E-22	↑^***^	
L-Acetylcarnitine	204.123	1.226	ESI+	2.71	1.16	1.49E-20	↑^***^	
O-Butanoylcarnitine	232.154	4.447	ESI+	3.20	1.14	1.72E-20	↑^***^	
O-Decanoyl-L-carnitine	316.248	6.134	ESI+	2.57	1.03	1.57E-10	↑^***^	
L-Palmitoylcarnitine	400.342	7.660	ESI+	1.95	1.05	1.20E-16	↑^***^	
Stearoylcarnitine	428.373	8.488	ESI+	1.91	1.08	8.90E-16	↑^***^	
Linoleyl carnitine	424.342	7.383	ESI+	2.23	1.06	5.90E-18	↑^***^	
Oleoylcarnitine	426.357	7.844	ESI+	2.06	1.12	4.37E-18	↑^***^	
LysoPC(O-18:0)	532.369	5.576	ESI+	2.12	0.73	6.28E-09	↑^***^	
SM d35:3	734.525	7.181	ESI+	1.66	1.08	1.31E-24	↑^***^	
PA(18:1(9Z)/22:4(7Z,10Z,13Z,16Z))	751.526	6.193	ESI+	2.19	1.27	1.31E-25	↑^***^	
PE(13:0/16:1(9Z))	648.453	5.991	ESI+	1.99	1.20	1.02E-22	↑^***^	
PE(14:1(9Z)/P-16:0)	646.473	7.285	ESI+	1.57	1.09	3.59E-08	↑^**^	
PS(P-16:0/12:0)	664.448	5.641	ESI+	2.11	1.00	8.12E-21	↑^***^	
LDGTS 7:0	348.238	4.793	ESI+	3.33	1.74	6.22E-20	↑^***^	

**p<0.01, ***p<0.001. CKD-SHPT group versus CKD group. CKD, chronic kidney disease; CKD-SHPT, chronic kidney disease complicated with secondary hyperparathyroidism; FC, fold change; SHPT, secondary hyperparathyroidism; VIP, variable importance in projection.

### 3.6 Correlation of differential metabolites with laboratory indices

To further clarify whether differential lipid metabolites were associated with decreased renal function and abnormal lipid levels in patients with CKD-SHPT, Spearman correlations were performed. All the differential lipid metabolites were correlated with conventional indices of renal function and blood lipid levels; they were positively correlated with urea, serum creatinine, cystatin C, and TG and negatively correlated with eGFR, TC, HDL-C, and LDL-C (**Table 4**).

**Table 4:** Correlation of lipid metabolites with clinical indicators of renal function and blood lipids.

Metabolites	Urea	SCr	CysC	eGFR	TC	TG	HDL-C	LDL-C
Carnitine	0.594, <0.001	0.725, <0.001	0.770, <0.001	-0.771, <0.001	-0.285, <0.001	0.203, 0.007	-0.226, <0.001	-0.337, <0.001
O-Butanoylcarnitine	0.706, <0.001	0.796, <0.001	0.819, <0.001	-0.825, <0.001	-0.286, <0.001	0.229, 0.001	-0.281, <0.001	-0.337, <0.001
O-Decanoyl-L-carnitine	0.510, <0.001	0.664, <0.001	0.668, <0.001	-0.697, <0.001	-0.286, <0.001	0.229, 0.001	-0.281, <0.001	-0.337, <0.001
L-Palmitoylcarnitine	0.519, <0.001	0.638, <0.001	0.693, <0.001	-0.691, <0.001	-0.303, <0.001	0.254, <0.001	-0.298, <0.001	-0.344, <0.001
jiStearoylcarnitine	0.489, <0.001	0.612, <0.001	0.665, <0.001	-0.677, <0.001	-0.346, 0.001	0.213, 0.001	-0.296, 0.001	-0.391, <0.001
Linoleyl carnitine	0.471, <0.001	0.632, <0.001	0.680, <0.001	-0.677, <0.001	-0.278, <0.001	0.273, <0.001	-0.330, <0.001	-0.306, <0.001
Oleoylcarnitine	0.541, <0.001	0.656, <0.001	0.700, <0.001	-0.710, <0.001	-0.375, 0.002	0.207, 0.002	-0.312, <0.001	-0.423, <0.001
LysoPC(O-18:0)	0.549, <0.001	0.658, <0.001	0.681, <0.001	-0.693, <0.001	-0.298, <0.001	0.264, 0.003	-0.361, <0.001	-0.292, <0.001
SM d35:3	0.441, <0.001	0.513, <0.001	0.517, <0.001	-0.537, <0.001	-0.218, 0.002	0.214, 0.003	-0.261, <0.001	-0.215, 0.003
PA(18:1(9Z)/22:4(7Z,10Z,13Z,16Z))	0.557, <0.001	0.669, <0.001	0.684, <0.001	-0.695, <0.001	-0.301, <0.001	0.239, 0.001	-0.315, <0.001	-0.312, <0.001
PE(13:0/16:1(9Z))	0.519, <0.001	0.625, <0.001	0.639, <0.001	-0.652, <0.001	-0.302, <0.001	0.278, 0.002	-0.343, <0.001	-0.320, <0.001
PE(14:1(9Z)/P-16:0)	0.411, <0.001	0.503, <0.001	0.495, <0.001	-0.524, <0.001	-0.192, 0.007	0.209, 0.003	-0.270, <0.001	-0.212, 0.003
PS(P-16:0/12:0)	0.578, <0.001	0.698, <0.001	0.701, <0.001	-0.708, <0.001	-0.337, <0.001	0.265, 0.003	-0.380, <0.001	-0.342, <0.001
LDGTS 7:0	0.620, <0.001	0.746, <0.001	0.797, <0.001	-0.792, <0.001	-0.396, <0.001	0.198, 0.013	-0.338, <0.001	-0.430, <0.001

Data are expressed as r, p. CysC, cystatin C; eGFR, estimated glomerular filtration rate; HDL-C, high-density lipoprotein cholesterol; LDL-C, low-density lipoprotein cholesterol; SCr, serum creatinine; TC, total cholesterol; TG, triglycerides.

## 4 Discussion

Few studies have examined the metabolic signature of SHPT. In the present study, we selected patients with CKD stages 3−5 and SHPT who were not receiving dialysis and attempted to understand the overall metabolic signature. We found that biotin and uridine levels were upregulated in SHPT, consistent with previous studies (11, 12). Notably, more differential metabolites were identified, including byproducts of tryptophan and phenylalanine metabolism. This indicates that disorders in amino acid metabolism caused by decreased biosynthesis or increased metabolism of tryptophan and phenylalanine occur in SHPT. Tryptophan and phenylalanine are essential amino acids, the metabolism of which is directly and indirectly regulated by intestinal microorganisms. Their metabolites are related to inflammation and immune, metabolic, and neuroregulatory functions, and have become therapeutic targets for various diseases. The concentration of tryptophan in human tissues is very low, as it is degraded by three main metabolic pathways: kynurenine (accounting for ~95% of tryptophan degradation), serotonin (~5%), and indole pathways. The kynurenine pathway is responsible for the *de novo* synthesis of NAD^+^, through an unstable intermediate that is easily converted to 2-picolinic acid, thereby limiting NAD^+^ production. Our study showed that patients with SHPT have increased levels of 2-picolinic acid, indicating restricted NAD^+^ synthesis. As the NAD^+^ level is an index of mitochondrial function (12), these findings suggest mitochondrial dysfunction in patients with SHPT.

Patients with SHPT and long-term high levels of PTH can experience damage to multiple systems, including the skeletal system. PTH acts on osteoclasts, causing severe bone pain, osteoporosis, bone collapse, deformity, periarticular lesions, and pathological fractures (20, 21). In this paper, we showed that phenylalanine, tyrosine, and tryptophan metabolic pathways are disturbed in patients with CKD-SHPT. It has been reported that some related metabolites of this pathway, such as kynurenic acid and 5-hydroxytryptophan, have a harmful effect on bone (22, 23), reduce bone mineral density, and increase fracture risk (24, 25). Otherwise, kynurenic acid is associated with bone marrow stromal cells and bone metabolism, and elevated kynurenic acid levels inhibit osteoblast metabolism through impaired mitochondrial respiration and reduce osteoblast numbers *in vivo* (26). This process also produces a large amount of reactive oxygen species, which can damage cells and inhibit the activity of osteoblasts. In addition, other metabolites have been reported to be associated with bone turnover. Indoxyl sulfate has been shown to decrease bone mineral density and increase fracture risk (25). The binding of 5-hydroxytryptophan to its receptor reduces the proliferation and differentiation of osteoblasts (23). The above metabolites were also significantly up-regulated in patients with CKD-SHPT in this study, suggesting that bone damage should be monitored in patients with SHPT to allow timely intervention.

Tryptophan can also be converted by the intestinal microbiota into indole and its derivatives to maintain intestinal homeostasis by regulating pro- and anti-inflammatory cytokines (27). Recent studies have revealed that PTH regulates bone mass through interactions with microbial metabolites, the immune system, and bone (28). Indoxyl sulfate is a pro-inflammatory metabolite that has nephrotoxic effects on renal proximal tubular cells (29), reducing bone formation, stimulating osteoblast apoptosis, and suppressing osteoclast differentiation (30). Indoxyl sulfate may also induce skeletal resistance to PTH in patients with CKD (31). Gut dysbiosis commonly develops in patients with CKD due to the accumulation of uremic toxins, dietary changes, frequent antibiotic therapy, altered intestinal mobility, and drug treatment (32, 33); accumulated uremic toxins may also exacerbate renal osteodystrophy in patients with SHPT. Uremic toxins accumulate throughout the body; those deposited in kidney tissue can increase oxidative stress and inflammatory cytokine production, thereby further promoting renal fibrosis (34). In this study, we found increased levels of uremic toxins in patients with SHPT.

Acylcarnitines, formed by the combination of fatty acids and carnitine, are associated with the β-oxidation of fatty acids. In addition to being valuable markers of inherited fatty acid metabolism disorders (35), they have also been linked to diabetes, cardiovascular disease, and cancer (36–38). Short-chain acylcarnitines are the most abundant, accounting for 80% or more of all acylcarnitines; L-acetylcarnitine is the most common. Long-chain acylcarnitines are mainly responsible for transporting long-chain fatty acids to the mitochondria for normal energy metabolism (39). In the present study, the levels of two short-chain acylcarnitines (L-acetylcarnitine and O-butanoylcarnitine), one medium-chain acylcarnitine (O-decanoyl-L-carnitine), and four long-chain acylcarnitines (L-palmitoylcarnitine, stearoylcarnitine, linoleyl carnitine, and oleoylcarnitine) were significantly higher in patients with SHPT than in those without and healthy controls, indicating obstruction of fatty acid oxidation and disordered mitochondrial metabolism. These results suggest that restoring normal endogenous fatty acid metabolism and mitochondrial oxidative phosphorylation may help slow CKD progression. In addition, long-chain acylcarnitines were more prevalent than short- and medium-chain acylcarnitines among the differential metabolites between patients with CKD with and without SHPT (with some not listed). Previous studies have shown that long-chain acylcarnitines may also contribute to the development of malignant ventricular arrhythmias in certain individuals (40, 41). Therefore, long-chain acylcarnitines may contribute to the increased incidence of cardiovascular events in patients with SHPT.

Increased levels of long-chain acylcarnitines in plasma is also an indicator of lipid metabolism disorder. Impaired fatty acid oxidation leads to disordered lipid metabolism, causing excessive deposition of various types of lipids, including free fatty acids, cholesterol, and phospholipids, in cells other than adipocytes, leading to lipotoxicity (42). In this study, the levels of several lipids were found to be increased in SHPT, indicating disordered lipid metabolism and deposition in patients with CKD and SHPT. Kang et al. (43) reported that the levels of key factors related to fatty acid oxidation and lipid deposition were low in the renal tubular epithelial cells of fibrotic human and mouse kidneys; the main deposited lipids were stearic, palmitoleic, and linoleic acids. This is consistent with the long-chain acylcarnitines found in the present study. Dysfunctional mitochondria are unable to process these long-chain fatty acids, leading to elevated plasma concentrations and deposition in renal mesangial cells, podocytes, and renal tubular epithelial cells. This further causes mitochondrial injury, oxidative stress, endoplasmic reticulum stress, and even renal cell apoptosis, eventually accelerating the progression of CKD in patients with SHPT.

Dysregulated lipid metabolism, which leads to dyslipidemia, has been reported in several kidney diseases. Uremia manifests as elevated levels of TG and LDL-C, and decreased levels of HDL-C (42); nephrotic syndrome is characterized by high levels of TC, TG, and LDL-C, and normal levels of HDL-C (44). This study showed no statistical differences in HDL-C and LDL-C levels between patients with SHPT and those without, suggesting that dyslipidemia in SHPT with CKD differs from that in uremia and nephrotic syndrome. Recent studies have reported that indoxyl sulfate can directly induce macrophage inflammation and impair cholesterol efflux to HDL, leading to the formation of foam cells and accelerating atherosclerosis in patients with CKD (45). This may explain the downregulated serum TC and HDL-C levels in patients with SHPT. Furthermore, correlation analysis showed that 14 lipid metabolites were significantly correlated with routine renal function and lipid indices, indicating that with the deterioration of renal function, the manifestations of disorders of lipid metabolism and dyslipidemia in the progression of CKD are complex.

Kyoto Encyclopedia of Genes and Genomes analysis revealed phenylalanine, tyrosine, and tryptophan biosynthesis as the metabolic pathway that differed most significantly between patients with and without SHPT. Nutrients from food can be metabolized by intestinal microorganisms to produce uremic toxins or their precursors. With decreased renal function in patients with CKD, these uremic toxins cannot be effectively removed and accumulate in the body. The predominance of bacterial families that produce the enzymes urease, uricase, indole, and p-cresol in patients with CKD (46) further increases the conversion of nutrients to uremic toxins, creating a vicious circle. These toxins accumulate in the circulation and damage tissues and organs throughout the body. Several studies have shown that uremic toxins produced in amino acid metabolism pathways are closely related to the progression of CKD (47–49), and therapeutic strategies targeting the intestinal microbiota to regulate gut-derived uremic toxins in patients with CKD have attracted considerable interest. Malnutrition in patients with CKD changes the balance between commensals and pathogens; the overgrowth of pathogenic bacteria can cause intestinal inflammation and loss of intestinal barrier function, allowing the transfer of bacterial components and even live bacteria. This induces an immune response leading to systemic inflammation. Amino acid metabolism plays important roles in adaptive and innate immunity, regulation of immune cell activation, and antibody production (50). Furthermore, amino acid sensing is associated with control of intestinal inflammation (51). In the present study, disturbances in phenylalanine, tyrosine, and tryptophan biosynthesis, and phenylalanine metabolism may be associated with increased systemic inflammation in SHPT.

This study has certain limitations. First, although the sample size of this study was larger than those of previous studies (11, 12), it was still small, meaning that subgroup analyses, for example according to CKD stage, could not be performed. Second, nutritional therapy is an important strategy to delay the progression of CKD, and most patients with CKD have restricted diets; therefore, the differential metabolites may reflect patients’ current nutritional and metabolic status. In addition, early treatment with medications such as phosphate binders and vitamin D analogs may have induced changes in metabolic pathways. Unfortunately, the treatment history of each patient was not recorded. Third, the differential metabolites identified lacked targeted validation. Future studies are therefore required to validate our findings and elucidate the metabolic characteristics of SHPT.

In summary, our study indicated that phenylalanine, tyrosine, and tryptophan biosynthesis was the most altered metabolic pathway in patients with CKD complicated by SHPT. Significant changes in amino acids, carnitines, and lipids were also observed; differential metabolites were correlated with aggravated renal function and abnormal blood lipid levels. For the first time, we outlined the metabolomic profile of patients with CKD stages 3−5 and SHPT who were not undergoing dialysis, which may provide a therapeutic foundation for the clinical management of SHPT.

## Data availability statement

The raw data supporting the conclusions of this article will be made available by the authors, without undue reservation.

## Ethics statement

The studies involving humans were approved by Ethical Review of Medical Technology committee Committee of Mianyang Central Hospita. The studies were conducted in accordance with the local legislation and institutional requirements. The participants provided their written informed consent to participate in this study.

## Author contributions

LG: Writing – original draft, Writing – review & editing, Conceptualization, Data curation, Investigation. LW: Writing – original draft, Writing – review & editing, Conceptualization, Data curation, Investigation. WL: Data curation, Formal analysis, Funding acquisition, Methodology, Software, Writing – review & editing, Conceptualization. YZ: Data curation, Formal analysis, Funding acquisition, Methodology, Software, Writing – review & editing, Conceptualization, Project administration. BX: Funding acquisition, Writing – original draft, Writing – review & editing, Conceptualization, Data curation, Formal analysis, Methodology, Project administration, Software.
