# Supplementary material for: Metabolomic profile of secondary hyperparathyroidism in patients with chronic kidney disease stages 3–5 not receiving dialysis
Source: Front Endocrinol (Lausanne). 2024 Jul 4;15:1406690. doi: 10.3389/fendo.2024.1406690 (PMC11254665; doi:10.3389/fendo.2024.1406690)
Supplement: Supplementary file 1 [file DataSheet_1.docx]

**
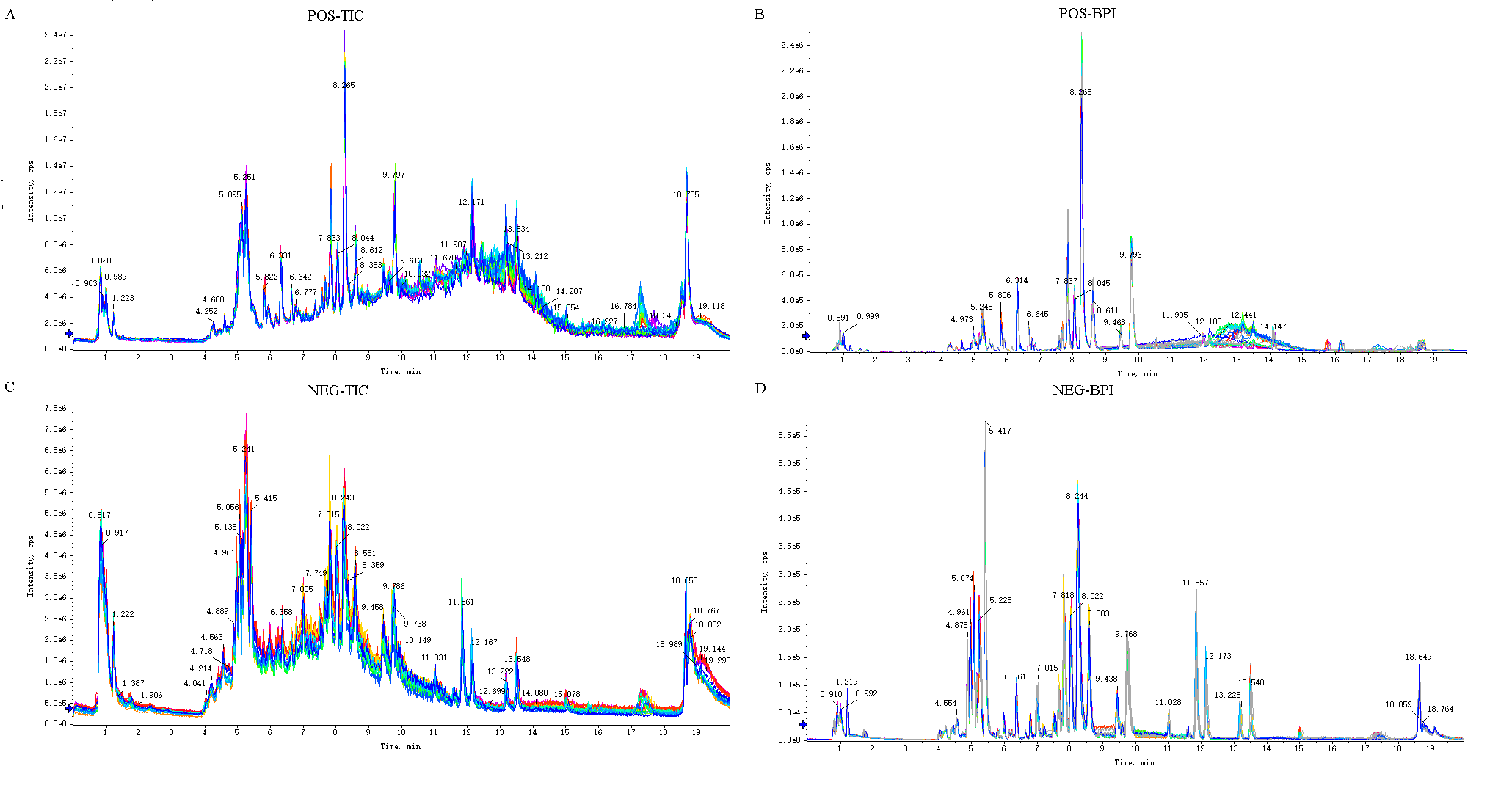
**

**Supplementary Figure S1** TIC and BPI diagrams of positive **(A, B)** and negative **(C, D)** ion modes. BPI, base peak intensity; TIC, total ion chromatogram.


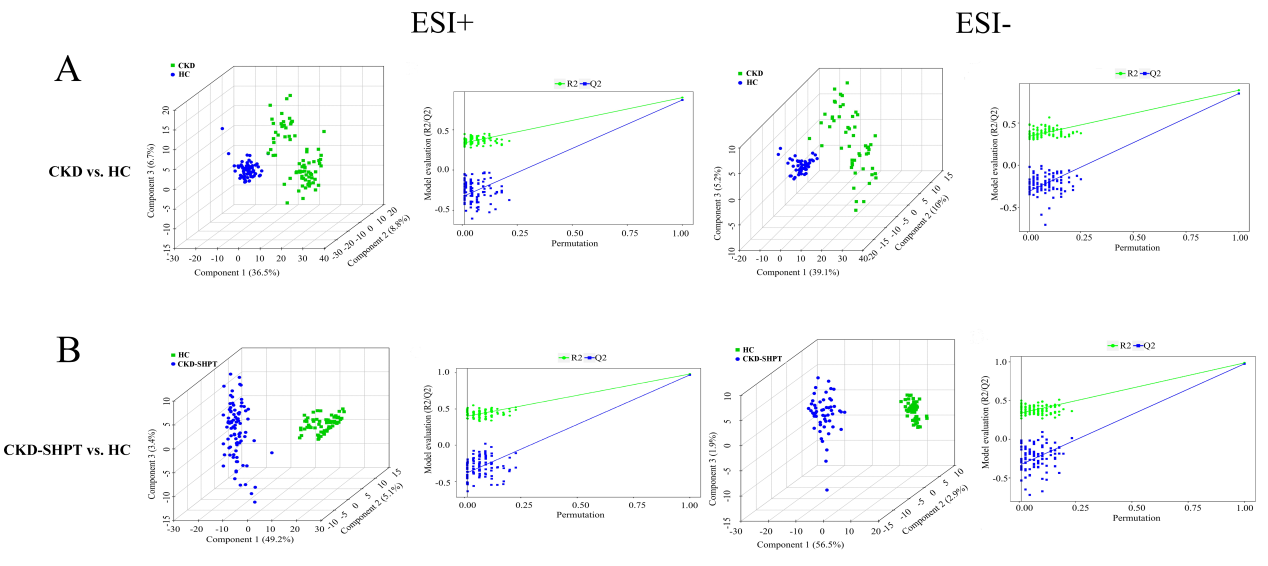


**Supplementary Figure S2** Plots of PLS-DA score and permutation testing for CKD vs. HC **(A)** and CKD-SHPT vs. HC **(B)** comparisons in ESI+ and ESI- scan modes. HC, healthy controls; CKD, chronic kidney disease; CKD-SHPT, chronic kidney disease complicated with secondary hyperparathyroidism; ESI, electrospray ionization; PLS-DA, partial least squares discriminant analysis.

| **Supplementary Table S1** Comparisons among groups under ESI+ and ESI− scan modes using PLS-DA analysis model. | | | | | |
| --- | --- | --- | --- | --- | --- |
| Scan mode | group | R^2^X_0_ | R^2^Y | Q^2^X_0_ | Q^2^Y |
| ESI+ | CKD vs.HC | 0.33 | 0.90 | -0.33 | 0.87 |
|  | CKD-SHPT  vs. HC | 0.39 | 0.98 | -0.40 | 0.97 |
| ESI− | CKD vs. HC | 0.34 | 0.89 | -0.32 | 0.85 |
|  | CKD-SHPT  vs. HC | 0.33 | 0.99 | -0.34 | 0.98 |

ESI, electrospray ionization; PLS-DA, partial least squares discriminant analysis. HC, healthy controls; CKD, chronic kidney disease; CKD-SHPT, chronic kidney disease complicated with secondary hyperparathyroidism.

| **Supplymentary Table S2** Significantly altered metabolic pathways in CKD vs HC and CKD-SHPT vs HC comparisons. | | | | | |
| --- | --- | --- | --- | --- | --- |
| Comparisons | Pathway Name | KEGG.id | -log（p） | Impact | Hits |
|  | Sphingolipid metabolism | hsa00600 | 0.64 | 0.43 | 4 |
| CKD vs. HC | Tryptophan metabolism | hsa00380 | 0.81 | 0.39 | 8 |
|  | Arginine biosynthesis | hsa00220 | 0.77 | 0.35 | 3 |
|  |  |  |  |  |  |
|  | D-Glutamine and D-glutamate metabolism | hsa00471 | 2.08 | 0.50 | 3 |
| CKD-SHPT vs. HC | Arginine biosynthesis | hsa00220 | 1.74 | 0.46 | 5 |
|  | Sphingolipid metabolism | hsa00600 | 0.34 | 0.43 | 4 |
|  | Glycerophospholipid metabolism | hsa00564 | 0.59 | 0.40 | 8 |
|  | Tryptophan metabolism | hsa00380 | 0.34 | 0.39 | 8 |
| HC, healthy controls; CKD, chronic kidney disease; CKD-SHPT, chronic kidney disease complicated with secondary hyperparathyroidism. | | | | | |
